# Algorithmic solutions for warehouse site selection using complex pythagorean fuzzy soft sets in supply chain management

**DOI:** 10.1038/s41598-025-02702-8

**Published:** 2025-08-29

**Authors:** Abaker A. Hassaballa, Ali Asghar, Tasadduq Niaz, Ines Hilali Jaghdam, Mohammad Sediq Safi

**Affiliations:** 1 Center for Scientific Research and Entrepreneurship, Northern Border University, Arar, 73213 Saudi Arabia; 2 Faculty of Sciences, Superior University Lahore, Sargodha Campus, Sargodha, 40100 Pakistan; 3 Department of Computer Science and Information Technology, Applied College, Princess Nourah bint Abdulrahman University, P.O. Box 84428, Riyadh, 11671 Saudi Arabia; 4 Department of Civil Engineering, Bakhtar University, Karte-char, Kabul, 15000 Afghanistan; 5 Department of Mathematics, Faculty of Science, Northern Border University, Arar, 73213 Saudi Arabia

**Keywords:** Pythagorean fuzzy soft set, Distance measures, Site selection, Decision-making, Supply chain management, Uncertainty, Mathematics and computing, Computational science

## Abstract

Global trade heavily depends on effective supply chain management and the strategic placement of distributors. Additionally, customer demands are becoming increasingly diverse and dynamic, with each customer expecting prompt and reliable responses from companies or distribution centers. To ensure a fast and efficient delivery process, businesses are striving to make well-informed decisions about the site selection of distribution centers or warehouses. The growth and success of online businesses, such as those on Amazon, largely hinge on the strategic site selection of their warehouses to expedite supply chain operations. This site selection process requires a comprehensive analysis of various factors. However, collecting and processing the relevant data often involves uncertainties and fuzziness. To address these challenges, the proposed research introduces a novel algorithmic approach based on distance measures within the Complex Pythagorean Fuzzy Soft Set (CPFSS) framework. The research presents the formulation of distance measures for the CPFSS, followed by the development of an algorithm. This algorithm is then applied to a real-life case study for the site selection of a warehouse for a company named HCRFT, which specializes in handicrafts. Furthermore, a detailed comparison between the proposed approach and existing models is conducted to validate and demonstrate the effectiveness of the algorithm. Finally, concluding remarks summarize the findings and implications of the study.

## Introduction

The world’s trade depends on the supply chain management and the location of distributors. Since world is moving to fast working in less consumption of time but to do this there is a problem related to the supply of different elements. For example, trading of surgical items, daily life use items, in different countries and then in any specific city of that country is very crucial because there are different well known companies which deals with the supply these item and are very less in number. Due to this reason, it is very difficult to make a well informed decision about the location of the distribution of that company in any specific city of one country. Many researcher^1–5^ worked to resolve this issue, the major problem is to provide a comprehensive algorithmic approach which can be used to select a feasible and reliable site selection of the distribution of any company in any city of a specific country. This complexity arises because only a few well-known companies manage the supply of these crucial items, making it difficult to make well-informed decisions about optimal distribution center locations within a city or country.

Since the classical set theory was unable to deal with uncertainty, therefore, to deal uncertainty in 1965, Zadeh^6^ extend the classical set to the fuzzy set (FS) in which elements are in the form of order pair such that first element represents the element of the universal set and the second is its membership value in the unit interval [0,1]. By introducing the non membership function, Atanassov^7^ defined the intuitionistic fuzzy set (IFS) in which elements are in the form of 3-tuples such that first element represents the element of the universal set, the second is its membership value in the unit interval [0,1] and third is its non-membership value in the unit interval [0,1] such that the sum of membership and non-membership remains in the interval [0,1]. Since there can be other situation that sum of membership and non-membership does not lie in the interval [0,1], therefore, to overcome this issue Yager^8^ introduced the Pythagorean fuzzy set (PFS) which extends the condition of IFS as sum of the square of membership and non-membership remains in the interval [0,1].

Since in the FS the membership function is real-valued function with range [0,1], therefore, Ramot et al.^9^ introduced the framework Complex Fuzzy Set (CFS) by defining complex valued membership function with range in the unit circle in FS. In the similar way, Alkouri et al.^10^ extended the concept of IFS to Complex Intuitionistic Fuzzy Set (CIFS) by defining the complex valued membership and non-membership functions such that sum of these two remains the unit circle. By continuing the advancement in the structures, Akram and Naz^11^ developed the structure of Complex Pythagorean Fuzzy Set (CPFS), in which sum of the square of complex valued membership and non-membership functions remains in the unit circle.

Molodtsov^12^ defined the Soft Set (SS) to deal the set of attributes with the universal in this set the elements are in the form of order pair in which first element represents the element of the attributes set and the second is a subset of the set of universal. By combining the both SS and the FS, Çaǧman et al.^13^ defined the innovative concept of the Fuzzy Soft Set (FSS) by using FS over the universal set in the definition of SS. By using IFS over the universal set in the definition of SS, Maji^14^ introduced the concept of the Intuitionistic Fuzzy Soft Set (IFSS). Further, the hybrid structure Pythagorean Fuzzy Soft Set (PFSS) is introduced by Peng et al.^23^, which is the combination of PFS and SS. Based on the discussion of earlier research, a structural comparison of the structures are shown in the Table 1, it is evident that the PFSS represents the latest advancement in SS theory.

**Table 1:** Structural Comparison.

Structure	Membership ($\nu$)	Non-Membership ($\omega$)	Amplitude	Phase	Condition
FS	$\checkmark$	$\times$	$\checkmark$	$\times$	$0\le \nu \le 1$
IFS	$\checkmark$	$\checkmark$	$\checkmark$	$\times$	$0\le \nu +\omega \le 1$
PFS	$\checkmark$	$\checkmark$	$\checkmark$	$\times$	$0\le \nu ^{2}+\omega ^{2} \le 1$
CFS	$\checkmark$	$\times$	$\checkmark$	$\checkmark$	$0\le \nu \le 1$
CIFS	$\checkmark$	$\checkmark$	$\checkmark$	$\checkmark$	$0\le \nu +\omega \le 1$
CPFS	$\checkmark$	$\checkmark$	$\checkmark$	$\checkmark$	$0\le \nu ^{2}+\omega ^{2} \le 1$

This study introduces the concept of the CPFSS, which extends the PFSS framework from a real-valued system to a complex-valued system. The PFSS structure offers a broad range of applications for decision-making experts, surpassing the capabilities of existing models discussed in the literature. Consequently, extending this structure to the complex domain through the CPFSS provides a more powerful tool for tackling complex decision-making problems. This article also explores the inclusion of a phase term in the CPFSS, demonstrating its utility for handling large datasets with potential repeating values.

CPFSS sets also gives a mathematical induction that combines the structure of PFSS and complex numbers. This generalization may lead to a more broad and demonstrative structure for the representation of uncertainty and fuzziness in real-life problems. The potential to design the complexity and ambiguity simultaneously can magnify the accuracy and applicability of this framework. The study of comparison with other existing models of FS and SS models can provide awareness into the superiority and limitations of CPFSS. Understanding how these models relate to and differ from each other can guide decision makers in choosing the most appropriate model for specific applications. This study provides a novel algorithmic framework for warehouse site selection by using the CPFSS with various distance measure. Unlike classical site selection models that specifically operate under the classic fuzzy sets, the proposed approach leverages the extended expressive power of CPFSS to model uncertainty, hesitation, and expert ambiguity more effectively. In addition, the use of various distance measures–such as Hamming, Euclidean, Hausdorff and Hellinger distances–enables a multidimensional analysis of alternatives. To the best of our knowledge, this is the first study to compare and aggregate such distance measures within the CPFSS setting for supply chain decision-making. The incorporation of a perturbation-based sensitivity analysis further adds to the robustness of the proposed method, offering new insights into the stability and adaptability of warehouse location decisions under data fluctuations. The selection of the CPFSS structure in this research is motivated by its superior capability to handle uncertainty and vagueness in expert evaluations, particularly in MCDM problems involving ambiguous or incomplete data. Since the FS and its extensions–such as IFS or PFS–allow for modeling degrees of membership and non-membership, they often fall short in capturing the phase or periodic nature of information that is common in real-world scenarios, such as logistics and supply chain planning. CPFSS combines the advantages of parameterization, Pythagorean fuzzy logic (greater expressive power than IFS), and complex numbers (modeling periodicity, direction, and oscillatory behavior), making it especially well-suited for contexts where data may have both quantitative uncertainty and qualitative vagueness. This richer structure allows decision-makers to more accurately represent expert judgment and uncertainty, leading to more nuanced and reliable outcomes.

In this paper, firstly the fundamentals of the CPFSS are introduced and also illustrated with the help of examples. Further four distance measures (Hamming, Euclidean, Hausdorff and Hellinger) between the CPFSS and the distance measures between the complex Pythagorean fuzzy soft elements are proposed with the proof of the distance measure properties. On the basis of the proposed distance measures, an algorithmic approach is also established which is then applied to the warehouse site selection case study for the validity and usefulness of the this approach. After the case study, the sensitivity analysis of the proposed model for the effectiveness of the model are presented. Further a comprehensive comparison of proposed algorithmic approach with existing models is presented. This comparison point-out the advancements of the presented model, specially in terms of its versatility, applicability and robustness in dealing with decision-making scenarios with complex data. Finally, this research is ended with a detailed concluding remarks which includes the future direction and limitations of the proposed approach.

## Fundamentals of complex pythagorean fuzzy soft set

### Preliminaries

Let’s recall some of the basic definitions from literature, which will be helpful in understanding the main concept of this article.

#### Definition 2.1

^6^ A fuzzy set over the set of universe $\breve{\mathbb {Q}}$ is defined as:

$$\mathfrak {F}_{\breve{\mathbb {Q}}}=\{(\Im , \digamma (\Im )): \Im \in \breve{\mathbb {Q}} \},$$

where $\digamma : \breve{\mathbb {Q}} \rightarrow [0,1]$ and $\digamma (\Im )$ is the membership degree of $\Im$.

#### Definition 2.2

^8^ A Pythagorean fuzzy set over the set of universe $\breve{\mathbb {Q}}$ is defined as:

$$\mathfrak {P}_{\breve{\mathbb {Q}}}=\bigg \{\big (\Im , \digamma (\Im ), \gimel (\Im )\big ): \Im \in \breve{\mathbb {Q}} \wedge \digamma , \gimel : \breve{\mathbb {Q}} \rightarrow [0,1] \bigg \},$$

where $\digamma (\Im )$ is the membership degree and $\gimel (\Im )$ is the non-membership degree of $\Im$ such that $(\digamma (\Im ))^{2}+(\gimel (\Im ))^{2}\le 1$.

#### Definition 2.3

^12^ For the set of universe $\breve{\mathbb {Q}}$ and for the subset $\mathbb {K}$ of set of parameters, consider the function $\grave{\mathcal {H}}:\mathbb {K}\rightarrow {\textbf {P}}(\breve{\mathbb {Q}})$, in which ${\textbf {P}}(\breve{\mathbb {Q}})$ represents the collection of all subset of $\breve{\mathbb {Q}}$. Then soft set is defined as:

$$\mathfrak {S}=\bigg \{\big (\varsigma ,\grave{\mathcal {H}}(\varsigma )\big ): \varsigma \in \mathbb {K} \wedge \grave{\mathcal {H}}(\varsigma )\in {\textbf {P}}(\breve{\mathbb {Q}}) \bigg \},$$

#### Definition 2.4

^23^ For the set of universe $\breve{\mathbb {Q}}$ and for the subset $\mathbb {K}$ of set of parameters, consider the function $\grave{\mathcal {H}}:\mathbb {K}\rightarrow \text {CPFs}(\breve{\mathbb {Q}})$, in which $\text {CPFs}(\breve{\mathbb {Q}})$ represents the collection of Pythagorean fuzzy subset overs $\breve{\mathbb {Q}}$. Then Pythagorean fuzzy soft set is defined as:

$$\mathfrak {P}=\bigg \{\big (\varsigma ,\grave{\mathcal {H}}(\varsigma )\big ): \varsigma \in \mathbb {K} \wedge \grave{\mathcal {H}}(\varsigma )\in \text {CPFs}(\breve{\mathbb {Q}}) \bigg \},$$

where $\grave{\mathcal {H}}(\varsigma )=\frac{\grave{\mathcal {H}}_{y}(\Im ),\grave{\mathcal {H}}_{n}(\Im )}{\Im },~\forall \Im \in \breve{\mathbb {Q}}$ with condition $0\le (\grave{\mathcal {H}}_{y}(\Im ))^{2}+(\grave{\mathcal {H}}_{n}(\Im ))^{2}\le 1$.

### Complex pythagorean fuzzy soft set and its examples

#### Definition 2.5

For the set of universe $\breve{\mathbb {Q}}$ and for the subset $\mathbb {K}$ of set of parameters, consider the function $\grave{\mathcal {H}}:\mathbb {K}\rightarrow \text {CPFs}(\breve{\mathbb {Q}})$, in which $\text {CPFs}(\breve{\mathbb {Q}})$ represents the collection of complex Pythagorean fuzzy subset overs $\breve{\mathbb {Q}}$. Then complex Pythagorean fuzzy soft set is defined as:

$$\mathfrak {P}_{y}=\bigg \{\big (\varsigma ,\grave{\mathcal {H}}(\varsigma )\big ): \varsigma \in \mathbb {K} \wedge \grave{\mathcal {H}}(\varsigma )\in \text {CPFs}(\breve{\mathbb {Q}}) \bigg \},$$

where $\grave{\mathcal {H}}(\varsigma )=\frac{\grave{\mathcal {H}}_{y}(\Im ),\grave{\mathcal {H}}_{n}(\Im )}{\Im },~\forall \Im \in \breve{\mathbb {Q}}$ such that $\grave{\mathcal {H}}_{y}(\Im )=\ddagger _{y}(\Im )e^{2i\pi (\natural _{y}(\Im ))}, \grave{\mathcal {H}}_{n}(\Im )=\ddagger _{n}(\Im )e^{2i\pi (\natural _{n}(\Im ))}$ with condition $0\le (\ddagger _{y}(\Im ))^{2}+(\ddagger _{n}(\Im ))^{2}\le 1,~0\le (\natural _{y}(\Im ))^{2}+(\natural _{n}(\Im ))^{2}\le 2\pi$.

#### Example 2.6

For the set of universe $\breve{\mathbb {Q}}=\{\Im _{1},\Im _{2},\Im _{3},\Im _{4}\}$ and for the subset $\mathbb {K}=\{ \varsigma _{1}, \varsigma _{3}, \varsigma _{5}, \varsigma _{6}\}$ of set of parameters $\{ \varsigma _{1}, \varsigma _{2}, \varsigma _{3}, \varsigma _{4}, \varsigma _{5}, \varsigma _{6}\}$, the approximate members of the set of CPFSS is given as:

$$\grave{\mathcal {H}}(\varsigma _{1})=\left\{ \begin{array}{l} \left( \Im _1, \langle 0.63\,e^{2\textbf{i}\pi (0.51)}, 0.41\,e^{2\textbf{i}\pi (0.52)}\rangle \right) ,\\ \left( \Im _2, \langle 0.52\,e^{2\textbf{i}\pi (0.62)}, 0.50\,e^{2\textbf{i}\pi (0.40)}\rangle \right) ,\\ \left( \Im _3, \langle 0.67\,e^{2\textbf{i}\pi (0.61)}, 0.41\,e^{2\textbf{i}\pi (0.41)}\rangle \right) ,\\ \left( \Im _4, \langle 0.59\,e^{2\textbf{i}\pi (0.46)}, 0.43\,e^{2\textbf{i}\pi (0.56)}\rangle \right) \end{array}\right\} ,$$

$$\grave{\mathcal {H}}(\varsigma _{3})=\left\{ \begin{array}{l} \left( \Im _1, \langle 0.53\,e^{2\textbf{i}\pi (0.49)}, 0.65\,e^{2\textbf{i}\pi (0.53)}\rangle \right) ,\\ \left( \Im _2, \langle 0.25\,e^{2\textbf{i}\pi (0.35)}, 0.79\,e^{2\textbf{i}\pi (0.70)}\rangle \right) ,\\ \left( \Im _3, \langle 0.70\,e^{2\textbf{i}\pi (0.72)}, 0.32\,e^{2\textbf{i}\pi (0.36)}\rangle \right) ,\\ \left( \Im _4, \langle 0.71\,e^{2\textbf{i}\pi (0.38)}, 0.39\,e^{2\textbf{i}\pi (0.63)}\rangle \right) \end{array}\right\} ,$$

$$\grave{\mathcal {H}}(\varsigma _{5})=\left\{ \begin{array}{l} \left( \Im _1, \langle 0.64\,e^{2\textbf{i}\pi (0.48)}, 0.41\,e^{2\textbf{i}\pi (0.53)}\rangle \right) ,\\ \left( \Im _2, \langle 0.23\,e^{2\textbf{i}\pi (0.65)}, 0.82\,e^{2\textbf{i}\pi (0.50)}\rangle \right) ,\\ \left( \Im _3, \langle 0.70\,e^{2\textbf{i}\pi (0.52)}, 0.34\,e^{2\textbf{i}\pi (0.46)}\rangle \right) ,\\ \left( \Im _4, \langle 0.28\,e^{2\textbf{i}\pi (0.16)}, 0.81\,e^{2\textbf{i}\pi (0.87)}\rangle \right) \end{array}\right\} ,$$

$$\grave{\mathcal {H}}(\varsigma _{6})=\left\{ \begin{array}{l} \left( \Im _1, \langle 0.43\,e^{2\textbf{i}\pi (0.54)}, 0.65\,e^{2\textbf{i}\pi (0.51)}\rangle \right) ,\\ \left( \Im _2, \langle 0.81\,e^{2\textbf{i}\pi (0.65)}, 0.22\,e^{2\textbf{i}\pi (0.50)}\rangle \right) ,\\ \left( \Im _3, \langle 0.70\,e^{2\textbf{i}\pi (0.52)}, 0.31\,e^{2\textbf{i}\pi (0.46)}\rangle \right) ,\\ \left( \Im _4, \langle 0.71\,e^{2\textbf{i}\pi (0.39)}, 0.36\,e^{2\textbf{i}\pi (0.63)}\rangle \right) \end{array}\right\} .$$

Rewrite the elements of the CPFSS in matrix form as:

$${\mathfrak {P}_{y}}_{1}=\left( {\begin{array}{*{20}{c}} \left\langle \begin{array}{l} \langle 0.63^{(0.51)}\\ 0.41^{(0.52)}\end{array}\right\rangle & \left\langle \begin{array}{l}\langle 0.52^{(0.62)}\\ 0.50^{(0.40)}\end{array}\right\rangle & \left\langle \begin{array}{l}\langle 0.67^{(0.61)}\\ 0.41^{(0.41)}\end{array}\right\rangle & \left\langle \begin{array}{l}\langle 0.59^{(0.46)}\\ 0.43^{(0.56)}\end{array}\right\rangle \\[10pt] \left\langle \begin{array}{l}\langle 0.53^{(0.49)}\\ 0.65^{(0.53)}\end{array}\right\rangle & \left\langle \begin{array}{l}\langle 0.25^{(0.35)}\\ 0.79^{(0.70)}\end{array}\right\rangle & \left\langle \begin{array}{l}\langle 0.70^{(0.72)}\\ 0.32^{(0.36)}\end{array}\right\rangle & \left\langle \begin{array}{l} \langle 0.71^{(0.38)}\\ 0.39^{(0.63)}\end{array}\right\rangle \\[10pt] \left\langle \begin{array}{l}\langle 0.64^{(0.48)}\\ 0.41^{(0.53)}\end{array}\right\rangle & \left\langle \begin{array}{l}\langle 0.23^{(0.65)}\\ 0.82^{(0.50)}\end{array}\right\rangle & \left\langle \begin{array}{l}\langle 0.70^{(0.52)}\\ 0.34^{(0.46)}\end{array}\right\rangle & \left\langle \begin{array}{l}\langle 0.28^{(0.16)}\\ 0.81^{(0.87)}\end{array}\right\rangle \\[10pt] \left\langle \begin{array}{l}\langle 0.43^{(0.54)}\\ 0.65^{(0.51)}\end{array}\right\rangle & \left\langle \begin{array}{l}\langle 0.81^{(0.65)}\\ 0.22^{(0.50)}\end{array}\right\rangle & \left\langle \begin{array}{l}\langle 0.70^{(0.52)}\\ 0.31^{(0.46)}\end{array}\right\rangle & \left\langle \begin{array}{l}\langle 0.71^{(0.39)}\\ 0.36^{(0.63)}\end{array}\right\rangle \end{array}} \right) .$$

Consider another CPFSS in matrix form as:

$${\mathfrak {P}_{y}}_{2}=\left( {\begin{array}{*{20}{c}} \left\langle \begin{array}{l} \langle 0.53^{(0.41)}\\ 0.51^{(0.62)}\end{array}\right\rangle & \left\langle \begin{array}{l}\langle 0.62^{(0.42)}\\ 0.40^{(0.50)}\end{array}\right\rangle & \left\langle \begin{array}{l}\langle 0.57^{(0.41)}\\ 0.51^{(0.52)}\end{array}\right\rangle & \left\langle \begin{array}{l}\langle 0.49^{(0.56)}\\ 0.53^{(0.46)}\end{array}\right\rangle \\[10pt] \left\langle \begin{array}{l}\langle 0.63^{(0.39)}\\ 0.55^{(0.43)}\end{array}\right\rangle & \left\langle \begin{array}{l}\langle 0.35^{(0.55)}\\ 0.69^{(0.50)}\end{array}\right\rangle & \left\langle \begin{array}{l}\langle 0.73^{(0.72)}\\ 0.36^{(0.39)}\end{array}\right\rangle & \left\langle \begin{array}{l} \langle 0.75^{(0.35)}\\ 0.49^{(0.53)}\end{array}\right\rangle \\[10pt] \left\langle \begin{array}{l}\langle 0.54^{(0.51)}\\ 0.61^{(0.56)}\end{array}\right\rangle & \left\langle \begin{array}{l}\langle 0.43^{(0.61)}\\ 0.72^{(0.42)}\end{array}\right\rangle & \left\langle \begin{array}{l}\langle 0.64^{(0.52)}\\ 0.54^{(0.46)}\end{array}\right\rangle & \left\langle \begin{array}{l}\langle 0.48^{(0.16)}\\ 0.56^{(0.87)}\end{array}\right\rangle \\[10pt] \left\langle \begin{array}{l}\langle 0.52^{(0.57)}\\ 0.49^{(0.51)}\end{array}\right\rangle & \left\langle \begin{array}{l}\langle 0.71^{(0.65)}\\ 0.32^{(0.52)}\end{array}\right\rangle & \left\langle \begin{array}{l}\langle 0.75^{(0.53)}\\ 0.33^{(0.41)}\end{array}\right\rangle & \left\langle \begin{array}{l}\langle 0.61^{(0.49)}\\ 0.52^{(0.45)}\end{array}\right\rangle \end{array}} \right) .$$

## The distance measures between complex pythagorean fuzzy soft sets

### Definition 3.1

For the set of universe $\breve{\mathbb {Q}}=\{\Im _{1},\Im _{2},\ldots ,\Im _{m}\}$ and for the subset $\mathbb {K}=\{ \varsigma _{1}, \varsigma _{2}, \ldots , \varsigma _{n}\}$ of set of parameters. Consider $\mathfrak {P}_{y1}=\bigg \{\big (\varsigma _{i},\grave{\mathcal {H}}_{1}(\varsigma _{i})\big ): \varsigma _{i}\in \mathbb {K} \wedge \grave{\mathcal {H}}_{1}(\varsigma )\in \text {CPFs}(\breve{\mathbb {Q}}) \bigg \},$ and $\mathfrak {P}_{y2}=\bigg \{\big (\varsigma _{i},\grave{\mathcal {H}}_{2}(\varsigma _{i})\big ): \varsigma _{i}\in \mathbb {K} \wedge \grave{\mathcal {H}}_{2}(\varsigma )\in \text {CPFs}(\breve{\mathbb {Q}}) \bigg \},$ are two CPFSSs, then the Hamming distance measure between two CPFSS is defined as:

1 $$\begin{aligned} & \mathfrak {D}^{1}(\mathfrak {P}_{y1},\mathfrak {P}_{y2})=\frac{1}{4mn}\sum _{j=1}^{m}\sum _{i=1}^{n}\bigg (\big |(\ddagger _{y1}^{i}(\Im _{j}))^{2}-(\ddagger _{y2}^{i}(\Im _{j}))^{2}\big |+ \big |(\ddagger _{n1}^{i}(\Im _{j}))^{2}-(\ddagger _{n2}^{i}(\Im _{j}))^{2}\big |\nonumber \\ & \quad +\big |(\natural _{y1}^{i}(\Im _{j}))^{2}-(\natural _{y2}^{i}(\Im _{j}))^{2}\big |+ \big |(\natural _{n1}^{i}(\Im _{j}))^{2}-(\natural _{n2}^{i}(\Im _{j}))^{2}\big |\bigg ), \end{aligned}$$

where $$\grave{\mathcal {H}}_{1}(\varsigma _{i})=\frac{\grave{\mathcal {H}}_{1y}^{i}(\Im ),\grave{\mathcal {H}}_{1n}^{i}(\Im )}{\Im }$$ and $$\grave{\mathcal {H}}_{2}(\varsigma _{i})=\frac{\grave{\mathcal {H}}_{2y}^{i}(\Im ),\grave{\mathcal {H}}_{2n}^{i}(\Im )}{\Im },~\forall \Im \in \breve{\mathbb {Q}}$$.

In addition, the Hamming distance measure between any two elements of CPFSS is defined as:

2 $$\begin{aligned} \mathfrak {D}_{e}^{1}(\mathfrak {P}_{y1},\mathfrak {P}_{y2})= & \frac{1}{4m}\sum _{j=1}^{m}\bigg (\big |(\ddagger _{y1}^{i}(\Im _{j}))^{2}-(\ddagger _{y2}^{i}(\Im _{j}))^{2}\big |+ \big |(\ddagger _{n1}^{i}(\Im _{j}))^{2}-(\ddagger _{n2}^{i}(\Im _{j}))^{2}\big |\nonumber \\ & +\big |(\natural _{y1}^{i}(\Im _{j}))^{2}-(\natural _{y2}^{i}(\Im _{j}))^{2}\big |+ \big |(\natural _{n1}^{i}(\Im _{j}))^{2}-(\natural _{n2}^{i}(\Im _{j}))^{2}\big |\bigg ), \end{aligned}$$

for any fixed *i*.

### Definition 3.2

For the set of universe $\breve{\mathbb {Q}}=\{\Im _{1},\Im _{2},\ldots ,\Im _{m}\}$ and for the subset $\mathbb {K}=\{ \varsigma _{1}, \varsigma _{2}, \ldots , \varsigma _{n}\}$ of set of parameters. Consider $\mathfrak {P}_{y1}=\bigg \{\big (\varsigma _{i},\grave{\mathcal {H}}_{1}(\varsigma _{i})\big ): \varsigma _{i}\in \mathbb {K} \wedge \grave{\mathcal {H}}_{1}(\varsigma )\in \text {CPFs}(\breve{\mathbb {Q}}) \bigg \},$ and $\mathfrak {P}_{y2}=\bigg \{\big (\varsigma _{i},\grave{\mathcal {H}}_{2}(\varsigma _{i})\big ): \varsigma _{i}\in \mathbb {K} \wedge \grave{\mathcal {H}}_{2}(\varsigma )\in \text {CPFs}(\breve{\mathbb {Q}}) \bigg \},$ are two CPFSSs, then the Euclidean distance measure between two CPFSS is defined as:

3 $$\begin{aligned} & \mathfrak {D}^{2}(\mathfrak {P}_{y1},\mathfrak {P}_{y2})=\bigg [\frac{1}{4mn}\sum _{j=1}^{m}\sum _{i=1}^{n}\bigg (\big |(\ddagger _{y1}^{i}(\Im _{j}))^{2}-(\ddagger _{y2}^{i}(\Im _{j}))^{2}\big |^{2}+ \big |(\ddagger _{n1}^{i}(\Im _{j}))^{2}-(\ddagger _{n2}^{i}(\Im _{j}))^{2}\big |^{2}\nonumber \\ & \quad +\big |(\natural _{y1}^{i}(\Im _{j}))^{2}-(\natural _{y2}^{i}(\Im _{j}))^{2}\big |^{2}+ \big |(\natural _{n1}^{i}(\Im _{j}))^{2}-(\natural _{n2}^{i}(\Im _{j}))^{2}\big |^{2}\bigg )\bigg ]^{\frac{1}{2}}, \end{aligned}$$

where $$\grave{\mathcal {H}}_{1}(\varsigma _{i})=\frac{\grave{\mathcal {H}}_{1y}^{i}(\Im ),\grave{\mathcal {H}}_{1n}^{i}(\Im )}{\Im }$$ and $$\grave{\mathcal {H}}_{2}(\varsigma _{i})=\frac{\grave{\mathcal {H}}_{2y}^{i}(\Im ),\grave{\mathcal {H}}_{2n}^{i}(\Im )}{\Im },~\forall \Im \in \breve{\mathbb {Q}}$$.

In addition, the Euclidean distance measure between any two elements of CPFSS is defined as:

4 $$\begin{aligned} \mathfrak {D}_{e}^{2}(\mathfrak {P}_{y1},\mathfrak {P}_{y2})= & \bigg [\frac{1}{4m}\sum _{j=1}^{m}\bigg (\big |(\ddagger _{y1}^{i}(\Im _{j}))^{2}-(\ddagger _{y2}^{i}(\Im _{j}))^{2}\big |^{2}+ \big |(\ddagger _{n1}^{i}(\Im _{j}))^{2}-(\ddagger _{n2}^{i}(\Im _{j}))^{2}\big |^{2}\nonumber \\ & \quad +\big |(\natural _{y1}^{i}(\Im _{j}))^{2}-(\natural _{y2}^{i}(\Im _{j}))^{2}\big |^{2}+ \big |(\natural _{n1}^{i}(\Im _{j}))^{2}-(\natural _{n2}^{i}(\Im _{j}))^{2}\big |^{2}\bigg )\bigg ]^{\frac{1}{2}}, \end{aligned}$$

for any fixed *i*.

### Definition 3.3

For the set of universe $\breve{\mathbb {Q}}=\{\Im _{1},\Im _{2},\ldots ,\Im _{m}\}$ and for the subset $\mathbb {K}=\{ \varsigma _{1}, \varsigma _{2}, \ldots , \varsigma _{n}\}$ of set of parameters. Consider $\mathfrak {P}_{y1}=\bigg \{\big (\varsigma _{i},\grave{\mathcal {H}}_{1}(\varsigma _{i})\big ): \varsigma _{i}\in \mathbb {K} \wedge \grave{\mathcal {H}}_{1}(\varsigma )\in \text {CPFs}(\breve{\mathbb {Q}}) \bigg \},$ and $\mathfrak {P}_{y2}=\bigg \{\big (\varsigma _{i},\grave{\mathcal {H}}_{2}(\varsigma _{i})\big ): \varsigma _{i}\in \mathbb {K} \wedge \grave{\mathcal {H}}_{2}(\varsigma )\in \text {CPFs}(\breve{\mathbb {Q}}) \bigg \},$ are two CPFSSs, then the Hausdorff distance measure between two CPFSS is defined as:

5 $$\begin{aligned} \mathfrak {D}^{3}(\mathfrak {P}_{y1},\mathfrak {P}_{y2})= & \frac{1}{2mn}\sum _{j=1}^{m}\sum _{i=1}^{n}\bigg (\max \big \{\big |(\ddagger _{y1}^{i}(\Im _{j}))^{2}-(\ddagger _{y2}^{i}(\Im _{j}))^{2}\big |, \big |(\ddagger _{n1}^{i}(\Im _{j}))^{2}-(\ddagger _{n2}^{i}(\Im _{j}))^{2}\big |\big \}\nonumber \\ & +\max \big \{\big |(\natural _{y1}^{i}(\Im _{j}))^{2}-(\natural _{y2}^{i}(\Im _{j}))^{2}\big |, \big |(\natural _{n1}^{i}(\Im _{j}))^{2}-(\natural _{n2}^{i}(\Im _{j}))^{2}\big |\big \}\bigg ), \end{aligned}$$

where $$\grave{\mathcal {H}}_{1}(\varsigma _{i})=\frac{\grave{\mathcal {H}}_{1y}^{i}(\Im ),\grave{\mathcal {H}}_{1n}^{i}(\Im )}{\Im }$$ and $$\grave{\mathcal {H}}_{2}(\varsigma _{i})=\frac{\grave{\mathcal {H}}_{2y}^{i}(\Im ),\grave{\mathcal {H}}_{2n}^{i}(\Im )}{\Im },~\forall \Im \in \breve{\mathbb {Q}}$$.

In addition, the Hausdorff distance measure between any two elements of CPFSS is defined as:

6 $$\begin{aligned} & \mathfrak {D}_{e}^{3}(\mathfrak {P}_{y1},\mathfrak {P}_{y2})=\frac{1}{2m}\sum _{j=1}^{m}\bigg (\max \big \{\big |(\ddagger _{y1}^{i}(\Im _{j}))^{2}-(\ddagger _{y2}^{i}(\Im _{j}))^{2}\big |, \big |(\ddagger _{n1}^{i}(\Im _{j}))^{2}-(\ddagger _{n2}^{i}(\Im _{j}))^{2}\big |\big \}\nonumber \\ & +\max \big \{\big |(\natural _{y1}^{i}(\Im _{j}))^{2}-(\natural _{y2}^{i}(\Im _{j}))^{2}\big |, \big |(\natural _{n1}^{i}(\Im _{j}))^{2}-(\natural _{n2}^{i}(\Im _{j}))^{2}\big |\big \}\bigg ), \end{aligned}$$

for any fixed *i*.

### Definition 3.4

For the set of universe $\breve{\mathbb {Q}}=\{\Im _{1},\Im _{2},\ldots ,\Im _{m}\}$ and for the subset $\mathbb {K}=\{ \varsigma _{1}, \varsigma _{2}, \ldots , \varsigma _{n}\}$ of set of parameters. Consider $\mathfrak {P}_{y1}=\bigg \{\big (\varsigma _{i},\grave{\mathcal {H}}_{1}(\varsigma _{i})\big ): \varsigma _{i}\in \mathbb {K} \wedge \grave{\mathcal {H}}_{1}(\varsigma )\in \text {CPFs}(\breve{\mathbb {Q}}) \bigg \},$ and $\mathfrak {P}_{y2}=\bigg \{\big (\varsigma _{i},\grave{\mathcal {H}}_{2}(\varsigma _{i})\big ): \varsigma _{i}\in \mathbb {K} \wedge \grave{\mathcal {H}}_{2}(\varsigma )\in \text {CPFs}(\breve{\mathbb {Q}}) \bigg \},$ are two CPFSSs, then the Hellinger distance measure between two CPFSS is defined as:

7 $$\begin{aligned} \mathfrak {D}^{4}(\mathfrak {P}_{y1},\mathfrak {P}_{y2})= & \bigg [\frac{1}{4mn}\sum _{j=1}^{m}\sum _{i=1}^{n}\bigg (\bigg (\sqrt{\ddagger _{y1}^{i}(\Im _{j})}-\sqrt{\ddagger _{y2}^{i}(\Im _{j})}\bigg )^{2}+ \bigg (\sqrt{\ddagger _{n1}^{i}(\Im _{j})}-\sqrt{\ddagger _{n2}^{i}(\Im _{j})}\bigg )^{2}\nonumber \\ & +\bigg (\sqrt{\natural _{y1}^{i}(\Im _{j})}-\sqrt{\natural _{y2}^{i}(\Im _{j})}\bigg )^{2}+ \bigg (\sqrt{\natural _{n1}^{i}(\Im _{j})}-\sqrt{\natural _{n2}^{i}(\Im _{j})}\bigg )^{2}\bigg )\bigg ]^{\frac{1}{2}}, \end{aligned}$$

where $$\grave{\mathcal {H}}_{1}(\varsigma _{i})=\frac{\grave{\mathcal {H}}_{1y}^{i}(\Im ),\grave{\mathcal {H}}_{1n}^{i}(\Im )}{\Im }$$ and $$\grave{\mathcal {H}}_{2}(\varsigma _{i})=\frac{\grave{\mathcal {H}}_{2y}^{i}(\Im ),\grave{\mathcal {H}}_{2n}^{i}(\Im )}{\Im },~\forall \Im \in \breve{\mathbb {Q}}$$.

In addition, the Hellinger distance measure between any two elements of CPFSS is defined as:

8 $$\begin{aligned} & \mathfrak {D}_{e}^{4}(\mathfrak {P}_{y1},\mathfrak {P}_{y2})=\bigg [\frac{1}{4m}\sum _{j=1}^{m}\bigg (\bigg (\sqrt{\ddagger _{y1}^{i}(\Im _{j})}-\sqrt{\ddagger _{y2}^{i}(\Im _{j})}\bigg )^{2}+ \bigg (\sqrt{\ddagger _{n1}^{i}(\Im _{j})}-\sqrt{\ddagger _{n2}^{i}(\Im _{j})}\bigg )^{2}\nonumber \\ & \quad +\bigg (\sqrt{\natural _{y1}^{i}(\Im _{j})}-\sqrt{\natural _{y2}^{i}(\Im _{j})}\bigg )^{2}+ \bigg (\sqrt{\natural _{n1}^{i}(\Im _{j})}-\sqrt{\natural _{n2}^{i}(\Im _{j})}\bigg )^{2}\bigg )\bigg ]^{\frac{1}{2}}, \end{aligned}$$

for any fixed *i*.

### Theorem 3.5

Consider that $\mathfrak {P}_{y1},\mathfrak {P}_{y2}, \mathfrak {P}_{y3}$ are CPFSSs, then functions $\mathfrak {D}^{p}(\mathfrak {P}_{ym},\mathfrak {P}_{yn})$ for $p=1,2,3,4$ and $m,n\in \{1,2,3\}$ must satisfy the following axioms:

1. $$\mathfrak {D}^{p}(\mathfrak {P}_{ym},\mathfrak {P}_{yn})\ge 0$$
2. $$\mathfrak {D}^{p}(\mathfrak {P}_{ym},\mathfrak {P}_{yn})=0 \iff \mathfrak {P}_{ym}=\mathfrak {P}_{yn}$$
3. $$\mathfrak {D}^{p}(\mathfrak {P}_{ym},\mathfrak {P}_{yn})=\mathfrak {D}^{p}(\mathfrak {P}_{yn},\mathfrak {P}_{ym})$$
4. $$\mathfrak {D}^{p}(\mathfrak {P}_{y1},\mathfrak {P}_{y3})\le \mathfrak {D}^{p}(\mathfrak {P}_{y1},\mathfrak {P}_{y2})+\mathfrak {D}^{p}(\mathfrak {P}_{y2},\mathfrak {P}_{y3}).$$

### Proof

To prove the all axioms for functions $\mathfrak {D}^{p}(\mathfrak {P}_{ym},\mathfrak {P}_{yn})$ for $p=1,2,3,4$ and $m,n\in \{1,2,3\}$, first consider the function

$$\begin{aligned} \mathfrak {D}^{1}(\mathfrak {P}_{y1},\mathfrak {P}_{y2})= & \frac{1}{4mn}\sum _{j=1}^{m}\sum _{i=1}^{n}\bigg (\big |(\ddagger _{y1}^{i}(\Im _{j}))^{2}-(\ddagger _{y2}^{i}(\Im _{j}))^{2}\big |+ \big |(\ddagger _{n1}^{i}(\Im _{j}))^{2}-(\ddagger _{n2}^{i}(\Im _{j}))^{2}\big |\\ & +\big |(\natural _{y1}^{i}(\Im _{j}))^{2}-(\natural _{y2}^{i}(\Im _{j}))^{2}\big |+ \big |(\natural _{n1}^{i}(\Im _{j}))^{2}-(\natural _{n2}^{i}(\Im _{j}))^{2}\big |\bigg ). \end{aligned}$$

*(1)* Since $|a-b|\ge 0$ for $a,b\in \mathbb {R}$ and sum of non-negative values is also non-negative, therefore,

$$\begin{aligned} & \frac{1}{4mn}\sum _{j=1}^{m}\sum _{i=1}^{n}\bigg (\big |(\ddagger _{y1}^{i}(\Im _{j}))^{2}-(\ddagger _{y2}^{i}(\Im _{j}))^{2}\big |+ \big |(\ddagger _{n1}^{i}(\Im _{j}))^{2}-(\ddagger _{n2}^{i}(\Im _{j}))^{2}\big |\\ & \quad +\big |(\natural _{y1}^{i}(\Im _{j}))^{2}-(\natural _{y2}^{i}(\Im _{j}))^{2}\big |+ \big |(\natural _{n1}^{i}(\Im _{j}))^{2}-(\natural _{n2}^{i}(\Im _{j}))^{2}\big |\bigg )\ge 0, \end{aligned}$$

$$\implies \mathfrak {D}^{1}(\mathfrak {P}_{y1},\mathfrak {P}_{y2})\ge 0.$$

*(2)* Consider that,

$$\begin{aligned} & \frac{1}{4mn}\sum _{j=1}^{m}\sum _{i=1}^{n}\bigg (\big |(\ddagger _{y1}^{i}(\Im _{j}))^{2}-(\ddagger _{y2}^{i}(\Im _{j}))^{2}\big |+ \big |(\ddagger _{n1}^{i}(\Im _{j}))^{2}-(\ddagger _{n2}^{i}(\Im _{j}))^{2}\big |\\ & \quad +\big |(\natural _{y1}^{i}(\Im _{j}))^{2}-(\natural _{y2}^{i}(\Im _{j}))^{2}\big |+ \big |(\natural _{n1}^{i}(\Im _{j}))^{2}-(\natural _{n2}^{i}(\Im _{j}))^{2}\big |\bigg )=0, \end{aligned}$$

Since in the left hand side, it is the sum of all non-negative values and it can be equal zero iff all the non-negative values are zero, therefore,

$$\big |(\ddagger _{y1}^{i}(\Im _{j}))^{2}-(\ddagger _{y2}^{i}(\Im _{j}))^{2}\big |=0, \big |(\ddagger _{n1}^{i}(\Im _{j}))^{2}-(\ddagger _{n2}^{i}(\Im _{j}))^{2}\big |=0,$$

$$\big |(\natural _{y1}^{i}(\Im _{j}))^{2}-(\natural _{y2}^{i}(\Im _{j}))^{2}\big |=0, \big |(\natural _{n1}^{i}(\Im _{j}))^{2}-(\natural _{n2}^{i}(\Im _{j}))^{2}\big |=0$$

Since $$|a-b|=0\iff a=b \forall a,b,\in \mathbb {R}$$, therefore,

$$(\ddagger _{y1}^{i}(\Im _{j}))^{2}=(\ddagger _{y2}^{i}(\Im _{j}))^{2}, (\ddagger _{n1}^{i}(\Im _{j}))^{2}=(\ddagger _{n2}^{i}(\Im _{j}))^{2}, (\natural _{y1}^{i}(\Im _{j}))=(\natural _{y2}^{i}(\Im _{j}))^{2}, (\natural _{n1}^{i}(\Im _{j}))^{2}=(\natural _{n2}^{i}(\Im _{j}))^{2}$$

By definition of CPFSS all the membership and non-membership functions are positive real valued functions with range [0, 1] and for all positive real numbers $a^{2}=b^{2}\iff a=b$, it follows that,

$$\ddagger _{y1}^{i}(\Im _{j})=\ddagger _{y2}^{i}(\Im _{j}), \ddagger _{n1}^{i}(\Im _{j})=\ddagger _{n2}^{i}(\Im _{j}), \natural _{y1}^{i}(\Im _{j})=\natural _{y2}^{i}(\Im _{j}), \natural _{n1}^{i}(\Im _{j})=\natural _{n2}^{i}(\Im _{j}),$$

which implies that $\mathfrak {P}_{ym}=\mathfrak {P}_{yn}$, it gives the required result.

*(3)* By using $|a-b|=|b-a|,~\forall a,b\in \mathbb {R}$, it can easily be proved that $\mathfrak {D}^{p}(\mathfrak {P}_{ym},\mathfrak {P}_{yn})=\mathfrak {D}^{p}(\mathfrak {P}_{yn},\mathfrak {P}_{ym}).$

*(4)* By using $|a-b|=|a-c+c-b|\le |a-c|+|c-b|,~\forall a,b,c\in \mathbb {R}$, the axiom $\mathfrak {D}^{p}(\mathfrak {P}_{y1},\mathfrak {P}_{y3})\le \mathfrak {D}^{p}(\mathfrak {P}_{y1},\mathfrak {P}_{y2})+\mathfrak {D}^{p}(\mathfrak {P}_{y2},\mathfrak {P}_{y3})$ can be proved easily.

In the similar way, the axioms for remaining functions $\mathfrak {D}^{p}(\mathfrak {P}_{ym},\mathfrak {P}_{yn})$ for $p=2,3,4$ can be proved. $\square$

## Application

This application section consist of three subsections, first with problem statement which need to be dealt, second with algorithmic approach which will be used to solve required problem and finally in the third subsection a daily life numerical problem is solved with help of the proposed algorithm.

### Problem statement

Global trade heavily relies on effective supply chain management and the strategic placement of distributors. As the world moves towards faster and more efficient operations, challenges related to the supply of essential goods have become increasingly evident. Additionally, customer demands are becoming more diverse and dynamic, with each customer expecting quick and reliable responses from companies or distribution centers. To meet these expectations, many companies have established logistics distribution networks to ensure efficient and timely service. The location of a logistics distribution center plays a critical role in meeting supply chain requirements effectively. However, many companies face significant challenges in determining optimal locations due to the ambiguity and uncertainty involved in the information required for site selection. Factors influencing distribution site selection are often complex and subject to various uncertainties, such as fluctuating demand patterns, transportation costs, and regional infrastructure. For example, in the field of e-commerce, customers expect prompt and reliable delivery when purchasing items across different countries and specific cities. To address these challenges, a well-informed decision-making approach is essential to handle the uncertainty and ambiguity inherent in the process. Developing a robust, data-driven model can help companies make strategic decisions about distribution center locations, ultimately enhancing efficiency and customer satisfaction in global trade.

### Algorithmic approach based on complex Pythagorean fuzzy soft set

**Figure 1:**
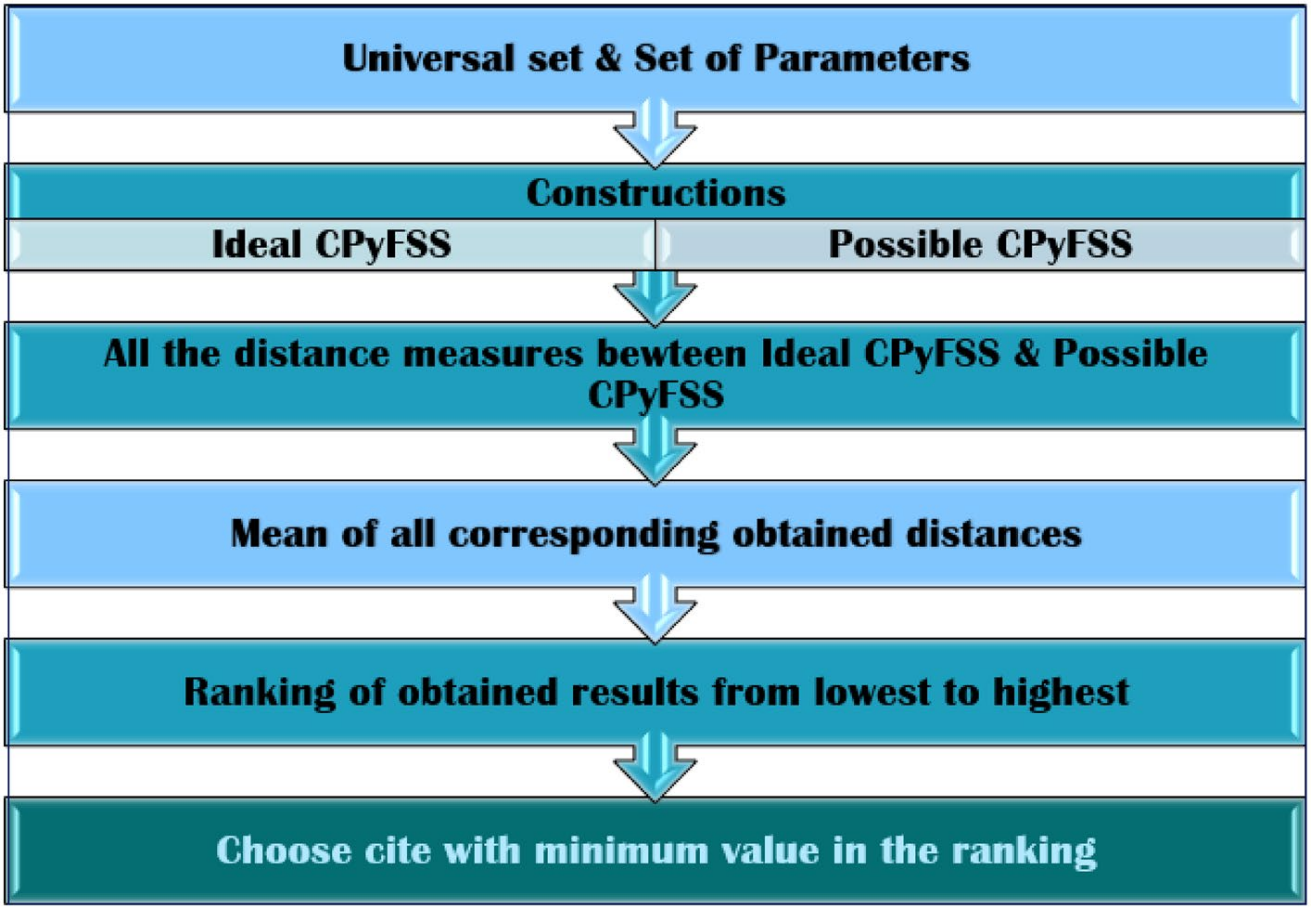
Algorithmic layout.

The algorithm based on the CPFSS consist of the following steps:

Step 1: Construction of the ideal CPFSS elements with the help of set of universe and by given set of parameters.
Step 2: For the given numerical problem, construct all the possible complex Pythagorean fuzzy soft elements with the help of set of universe and by given set of parameters.
Step 3: Find the distance between corresponding elements of the sets by using distance measure function.
Step 4: Calculate the mean for distances of elements of each set.
Step 5: Rank the obtained results from lowest to highest based on the mean of the distance measures.
Step 6: Choose the location with minimum ranking as best option from available options. The complete layout of the proposed algorithm is shown in Fig. 1.

### Metric appropriateness by criterion type

It is important to note that the appropriateness of a distance measure may depend on the nature of each evaluation criterion. For instance, Euclidean and Hellinger distances are more suitable for continuous and quantitative variables such as transportation cost or delivery time. On the other hand, Hamming distance may be more appropriate for categorical or binary-type attributes. In this study, we apply each metric uniformly across all factors to evaluate and compare their performance in the context of CPFSS. However, future work could explore adaptive or hybrid approaches, where metric selection is aligned with the data type of each criterion to potentially enhance the robustness of the decision-making process.

### Case study

**Figure 2:**
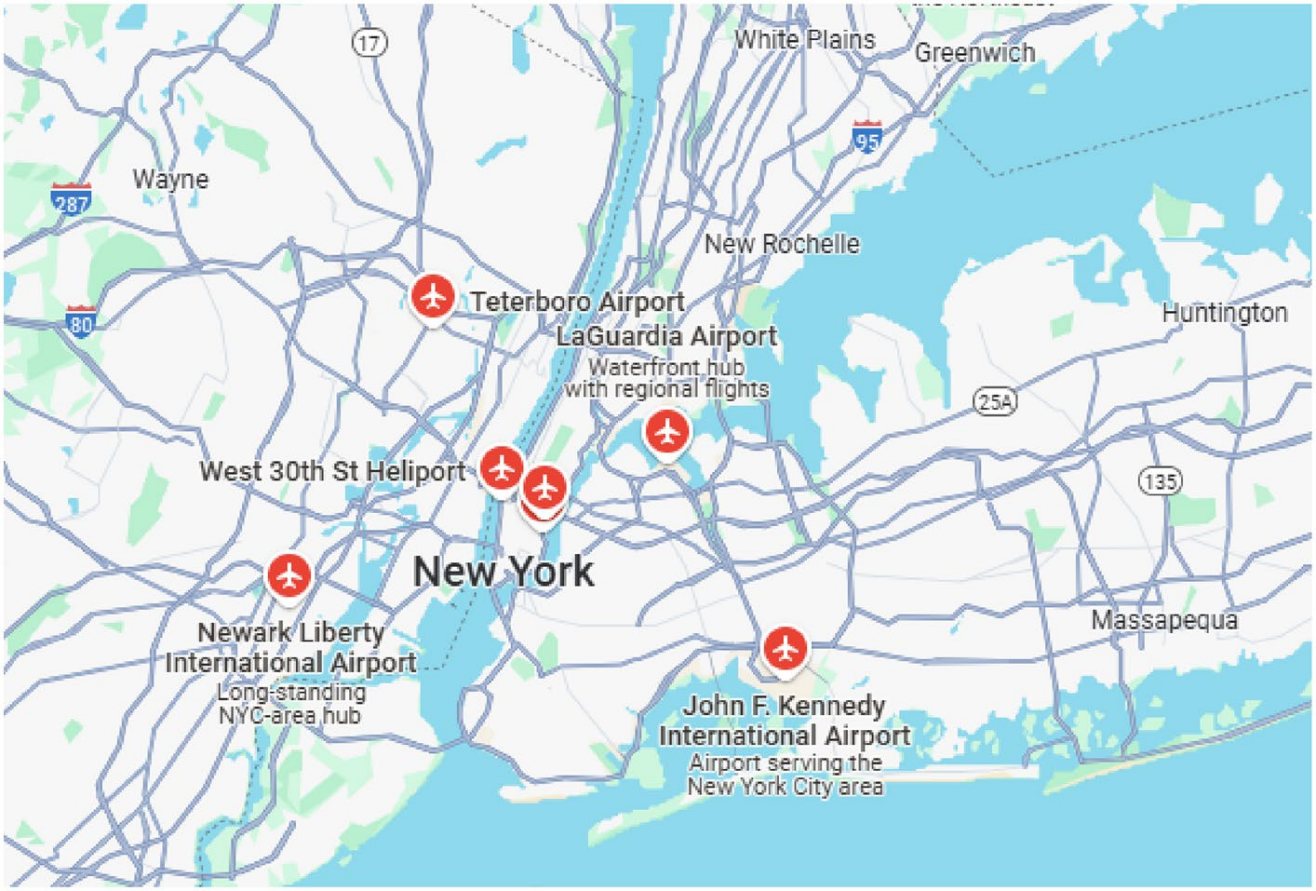
This map is showing locations of airports near New York city and it is generated using onlineMapTiler platform (https://www.maptiler.com/), version: 14.0.3.

**Figure 3:**
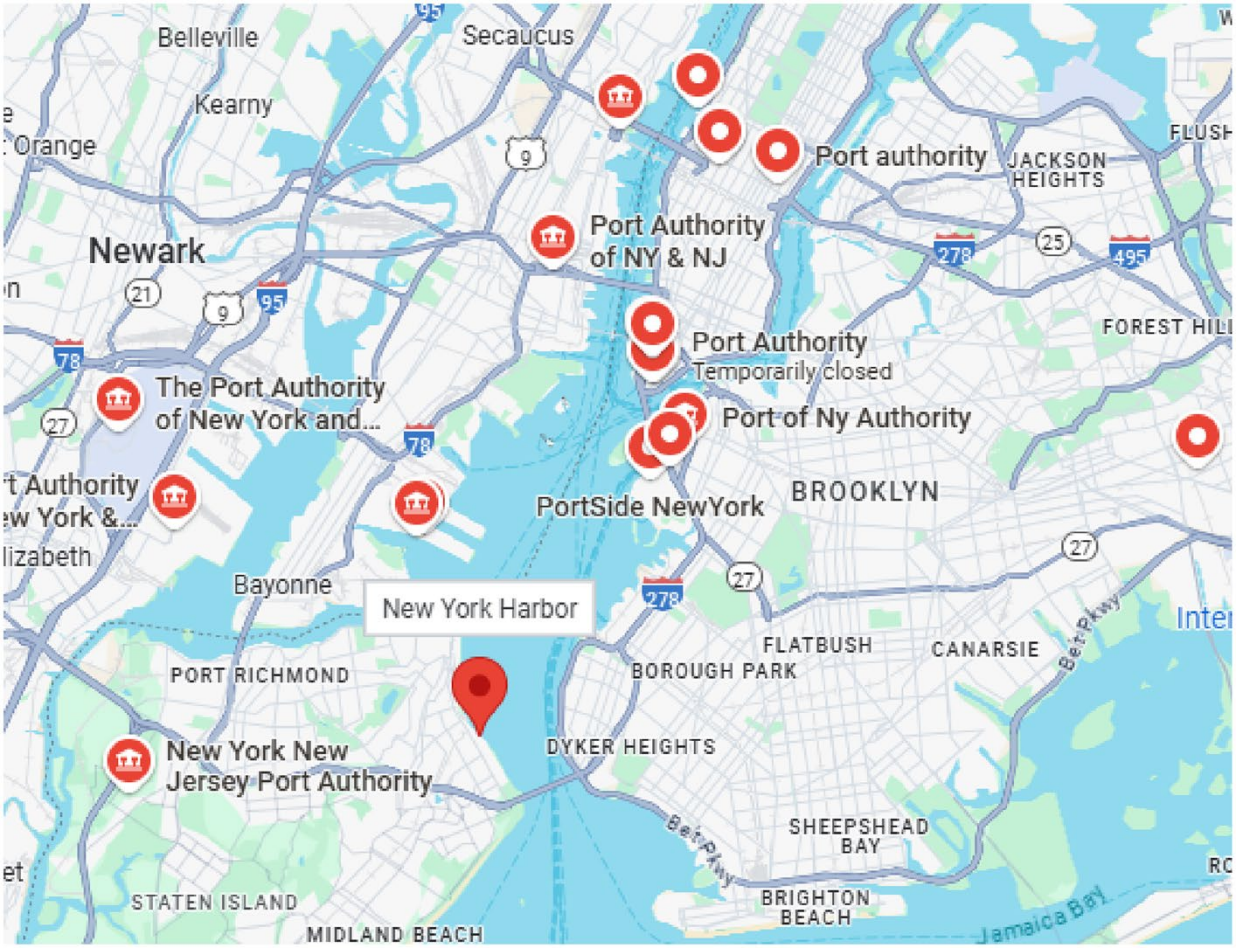
This map is showing locations of ports near New York city and it is generated using onlineMapTiler platform (https://www.maptiler.com/), version: 14.0.3.

A Pakistani company, HCRFT (a hypothetical name), specializing in handicrafts, plans to open an online store on Amazon to expand its sales in the USA, with the goal of further extending to other countries. According to Amazon’s policy, the company is required to establish a warehouse in the target country, which in this case is the USA. Consequently, the company’s management has decided to construct a warehouse in New York City. To achieve this, the company’s manager needs to make an informed decision regarding the site selection for the warehouse. Based on expert opinions, the selection depends on the following factors: Distance from the airport $(\Im _{1})$, distance from the port $(\Im _{2})$, delivery time $(\Im _{3})$, history of customer demand $(\Im _{4})$, and financial reliability $(\Im _{5})$. Considering these parameters, the experts have recommended four potential locations, denoted as $\varsigma _{1}, \varsigma _{2}, \varsigma _{3},$ and $\varsigma _{4}$. To select the most suitable site, let the set $\{\Im _{1}, \Im _{2}, \Im _{3}, \Im _{4}, \Im _{5}\}$ represent the universal set corresponding to the set of locations $\{\varsigma _{1}, \varsigma _{2}, \varsigma _{3}, \varsigma _{4}\}$ as the attribute set. To apply the proposed algorithm, data related to the influencing factors is required for these locations. This data will then be transformed into a complex Pythagorean fuzzy soft set with the assistance of experts. For distance-related data, such as proximity to the airport and port, maps of New York City (provided in Figs. 2 and 3) can be utilized.

Step 1: According to the universal set $\{\Im _{1},\Im _{2}, \Im _{3},\Im _{4},\Im _{5}\}$ and experts opinions, the complex Pythagorean fuzzy soft set for ideal location say $\varsigma$ is constructed, which is given as:
  $$\grave{\mathcal {H}}(\varsigma )=\left\{ \begin{array}{l} \left( \Im _1, \langle 0.88\,e^{2\textbf{i}\pi (0.51)}, 0.37\,e^{2\textbf{i}\pi (0.52)}\rangle \right) ,\\ \left( \Im _2, \langle 0.93\,e^{2\textbf{i}\pi (0.78)}, 0.30\,e^{2\textbf{i}\pi (0.34)}\rangle \right) ,\\ \left( \Im _3, \langle 0.89\,e^{2\textbf{i}\pi (0.81)}, 0.33\,e^{2\textbf{i}\pi (0.41)}\rangle \right) ,\\ \left( \Im _4, \langle 0.91\,e^{2\textbf{i}\pi (0.76)}, 0.29\,e^{2\textbf{i}\pi (0.36)}\rangle \right) ,\\ \left( \Im _5, \langle 0.89\,e^{2\textbf{i}\pi (0.85)}, 0.37\,e^{2\textbf{i}\pi (0.28)}\rangle \right) \end{array}\right\} ,$$
Step 2: According to the parameters $\{\Im _{1},\Im _{2}, \Im _{3},\Im _{4},\Im _{5}\}$ and experts opinions, the complex Pythagorean fuzzy soft set for available locations $\{\varsigma _{1},\varsigma _{2},\varsigma _{3},\varsigma _{4}\}$ are constructed, which are given as:
  $$\grave{\mathcal {H}}(\varsigma _{1})=\left\{ \begin{array}{l} \left( \Im _1, \langle 0.63\,e^{2\textbf{i}\pi (0.51)}, 0.41\,e^{2\textbf{i}\pi (0.52)}\rangle \right) ,\\ \left( \Im _2, \langle 0.62\,e^{2\textbf{i}\pi (0.62)}, 0.38\,e^{2\textbf{i}\pi (0.39)}\rangle \right) ,\\ \left( \Im _3, \langle 0.77\,e^{2\textbf{i}\pi (0.61)}, 0.41\,e^{2\textbf{i}\pi (0.47)}\rangle \right) ,\\ \left( \Im _4, \langle 0.69\,e^{2\textbf{i}\pi (0.57)}, 0.53\,e^{2\textbf{i}\pi (0.46)}\rangle \right) ,\\ \left( \Im _5, \langle 0.65\,e^{2\textbf{i}\pi (0.63)}, 0.43\,e^{2\textbf{i}\pi (0.56)}\rangle \right) \end{array}\right\} ,$$
  $$\grave{\mathcal {H}}(\varsigma _{2})=\left\{ \begin{array}{l} \left( \Im _1, \langle 0.63\,e^{2\textbf{i}\pi (0.51)}, 0.41\,e^{2\textbf{i}\pi (0.52)}\rangle \right) ,\\ \left( \Im _2, \langle 0.64\,e^{2\textbf{i}\pi (0.72)}, 0.46\,e^{2\textbf{i}\pi (0.40)}\rangle \right) ,\\ \left( \Im _3, \langle 0.59\,e^{2\textbf{i}\pi (0.69)}, 0.50\,e^{2\textbf{i}\pi (0.44)}\rangle \right) ,\\ \left( \Im _4, \langle 0.63\,e^{2\textbf{i}\pi (0.57)}, 0.51\,e^{2\textbf{i}\pi (0.56)}\rangle \right) ,\\ \left( \Im _5, \langle 0.59\,e^{2\textbf{i}\pi (0.51)}, 0.47\,e^{2\textbf{i}\pi (0.53)}\rangle \right) \end{array}\right\} ,$$
  $$\grave{\mathcal {H}}(\varsigma _{3})=\left\{ \begin{array}{l} \left( \Im _1, \langle 0.63\,e^{2\textbf{i}\pi (0.51)}, 0.41\,e^{2\textbf{i}\pi (0.52)}\rangle \right) ,\\ \left( \Im _2, \langle 0.71\,e^{2\textbf{i}\pi (0.67)}, 0.38\,e^{2\textbf{i}\pi (0.43)}\rangle \right) ,\\ \left( \Im _3, \langle 0.63\,e^{2\textbf{i}\pi (0.53)}, 0.47\,e^{2\textbf{i}\pi (0.49)}\rangle \right) ,\\ \left( \Im _4, \langle 0.58\,e^{2\textbf{i}\pi (0.51)}, 0.52\,e^{2\textbf{i}\pi (0.61)}\rangle \right) ,\\ \left( \Im _5, \langle 0.73\,e^{2\textbf{i}\pi (0.53)}, 0.45\,e^{2\textbf{i}\pi (0.66)}\rangle \right) \end{array}\right\} ,$$
  $$\grave{\mathcal {H}}(\varsigma _{4})=\left\{ \begin{array}{l} \left( \Im _1, \langle 0.63\,e^{2\textbf{i}\pi (0.51)}, 0.41\,e^{2\textbf{i}\pi (0.52)}\rangle \right) ,\\ \left( \Im _2, \langle 0.52\,e^{2\textbf{i}\pi (0.62)}, 0.50\,e^{2\textbf{i}\pi (0.40)}\rangle \right) ,\\ \left( \Im _3, \langle 0.67\,e^{2\textbf{i}\pi (0.61)}, 0.41\,e^{2\textbf{i}\pi (0.41)}\rangle \right) ,\\ \left( \Im _4, \langle 0.59\,e^{2\textbf{i}\pi (0.46)}, 0.43\,e^{2\textbf{i}\pi (0.56)}\rangle \right) ,\\ \left( \Im _5, \langle 0.59\,e^{2\textbf{i}\pi (0.46)}, 0.43\,e^{2\textbf{i}\pi (0.56)}\rangle \right) \end{array}\right\} ,$$
Step 3: Applied the proposed distance between the elements of CPFSS and their results are shown in Table 2:
Step 4: The calculated mean of the distances of each element is given in Table 3.
Step 5: From the step 4 values, the ranking of the distance measures is:
  $$\mathfrak {D}(\grave{\mathcal {H}}(\varsigma ),\grave{\mathcal {H}}(\varsigma _{1}))\le \mathfrak {D}(\grave{\mathcal {H}}(\varsigma ),\grave{\mathcal {H}}(\varsigma _{3})) \le \mathfrak {D}(\grave{\mathcal {H}}(\varsigma ),\grave{\mathcal {H}}(\varsigma _{2})) \le \mathfrak {D}(\grave{\mathcal {H}}(\varsigma ),\grave{\mathcal {H}}(\varsigma _{4}))$$
Step 6: Since the mean value of the distance $\mathfrak {D}(\grave{\mathcal {H}}(\varsigma ),\grave{\mathcal {H}}(\varsigma _{1}))$ is minimum, therefore, the best option for the warehouse is site $\varsigma _{1}$.

**Table 2:** Distance Measures.

Distances	$\mathfrak {D}(\grave{\mathcal {H}}(\varsigma ),\grave{\mathcal {H}}(\varsigma _{1}))$	$\mathfrak {D}(\grave{\mathcal {H}}(\varsigma ),\grave{\mathcal {H}}(\varsigma _{2}))$	$\mathfrak {D}(\grave{\mathcal {H}}(\varsigma ),\grave{\mathcal {H}}(\varsigma _{3}))$	$\mathfrak {D}(\grave{\mathcal {H}}(\varsigma ),\grave{\mathcal {H}}(\varsigma _{4}))$
$\mathfrak {D}_{e}^{1}$	1.3830	1.4872	1.7401	1.7852
$\mathfrak {D}_{e}^{2}$	0.8645	0.9328	0.4637	0.5691
$\mathfrak {D}_{e}^{3}$	1.5092	1.8337	1.5682	1.6889
$\mathfrak {D}_{e}^{4}$	0.7359	0.6710	0.8283	0.9718

**Table 3:** Mean of the distance measures.

Distances	$\mathfrak {D}(\grave{\mathcal {H}}(\varsigma ),\grave{\mathcal {H}}(\varsigma _{1}))$	$\mathfrak {D}(\grave{\mathcal {H}}(\varsigma ),\grave{\mathcal {H}}(\varsigma _{2}))$	$\mathfrak {D}(\grave{\mathcal {H}}(\varsigma ),\grave{\mathcal {H}}(\varsigma _{3}))$	$\mathfrak {D}(\grave{\mathcal {H}}(\varsigma ),\grave{\mathcal {H}}(\varsigma _{4}))$
Mean	1.12315	1.2312	1.1501	1.25375

### Rank divergence analysis

Note that before aggregating the results from all four distance measures, the individual rankings of the alternatives obtained using each measure is presented in Table 2. This breakdown helps to clarify the role and influence of each distance measure in shaping the final ranking. In addition, to evaluate the divergence among the rankings produced by different distance metrics, the Spearman’s rank correlation coefficients between each pair is calculated. Lower correlation values indicate greater divergence and suggest that the corresponding metric brings a distinct perspective to the decision-making process. The results are shown in Table 4.

**Table 4:** Spearman’s rank correlation coefficients between distance measures.

	$\mathfrak {D}_e^1$	$\mathfrak {D}_e^2$	$\mathfrak {D}_e^3$	$\mathfrak {D}_e^4$
$\mathfrak {D}_e^1$	1.000	0.800	0.800	0.948
$\mathfrak {D}_e^2$	0.800	1.000	0.800	0.400
$\mathfrak {D}_e^3$	0.800	0.800	1.000	0.632
$\mathfrak {D}_e^4$	0.948	0.400	0.632	1.000

According to the Table 4, the highest agreement is between $\mathfrak {D}_e^1$ and $\mathfrak {D}_e^4$ ($\rho$ = 0.948), which implies very strong similarity in rankings. The most divergence is between $\mathfrak {D}_e^2$ and $\mathfrak {D}_e^4$ ($\rho$ = 0.400), which implies moderate disagreement. This shows that $\mathfrak {D}_e^2$ contributes the most distinct perspective to the final aggregation.

## Sensitivity analysis

For the sensitivity and stability analysis of the proposed algorithm, various statistical tools, such as the geometric mean, harmonic mean, variance, and standard deviation, were employed instead of the arithmetic mean. By incorporating variance, standard deviation, sample variance, and sample standard deviation in the proposed algorithm, the results are presented in Table 5.

**Table 5:** Sensitivity and Stability Analysis.

Tools	$\mathfrak {D}(\grave{\mathcal {H}}(\varsigma ),\grave{\mathcal {H}}(\varsigma _{1}))$	$\mathfrak {D}(\grave{\mathcal {H}}(\varsigma ),\grave{\mathcal {H}}(\varsigma _{2}))$	$\mathfrak {D}(\grave{\mathcal {H}}(\varsigma ),\grave{\mathcal {H}}(\varsigma _{3}))$	$\mathfrak {D}(\grave{\mathcal {H}}(\varsigma ),\grave{\mathcal {H}}(\varsigma _{4}))$
Standard Deviation	0.3292	0.4559	0.5238	0.5050
Variance	0.1084	0.2078	0.2744	0.2550
Sample Standard Deviation	0.3801	0.5264	0.6048	0.5831
Sample Variance	0.1445	0.2771	0.3658	0.3400

**Figure 4:**
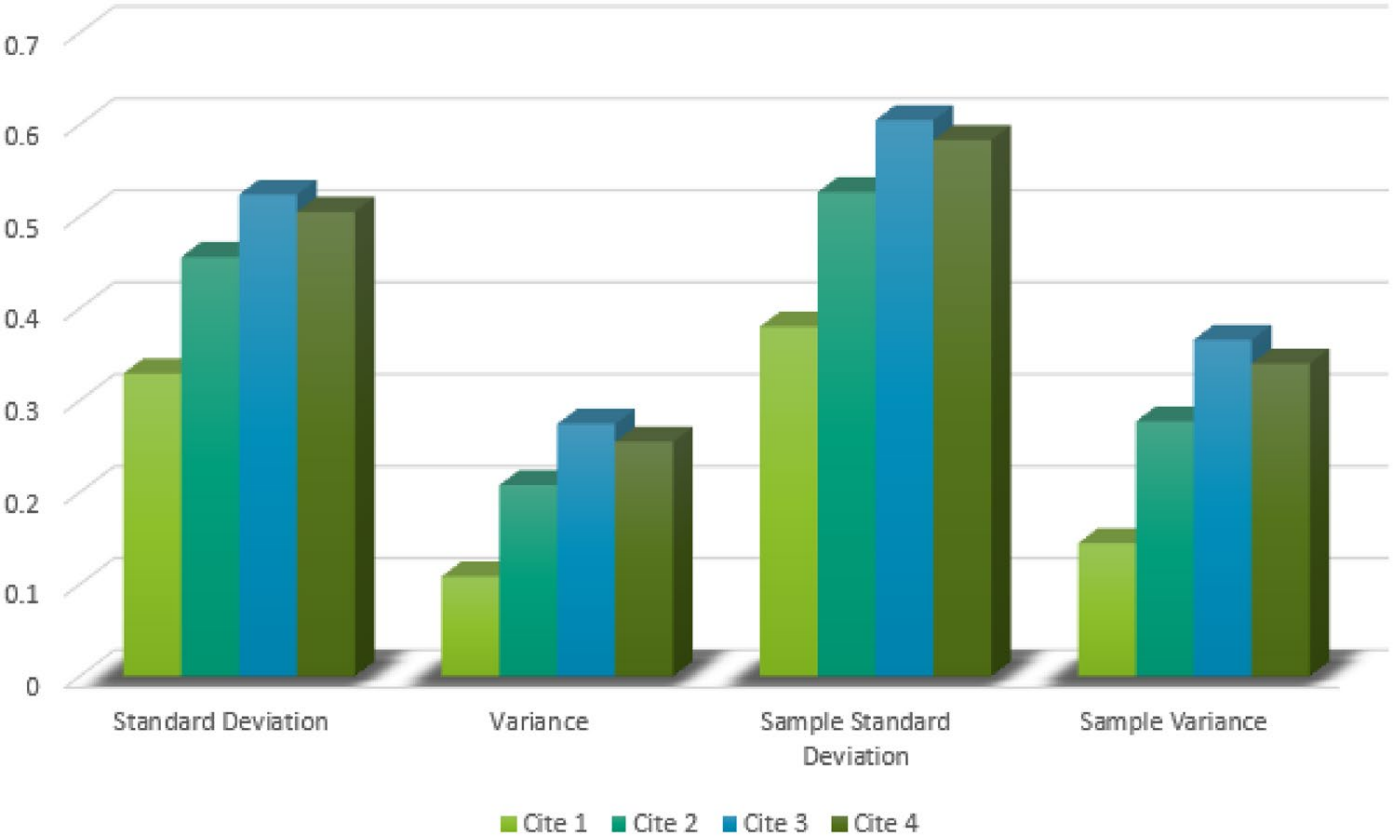
Stability analysis.

From Table 5, it is evident that the distance $\mathfrak {D}(\grave{\mathcal {H}}(\varsigma ),\grave{\mathcal {H}}(\varsigma _{1}))$ with respect to these statistical measures is minimized. This indicates that the algorithm performs consistently across different statistical measures, thereby ensuring its robustness. According to the proposed algorithm, the optimal site location is $\varsigma _{1}$. Furthermore, the consistency of results across different measures highlights the reliability of the algorithm in practical applications. This result aligns with the proposed algorithm, demonstrating its sensitivity and stability. The graphical representation of the stability and sensitivity analysis is presented in Fig. 4, demonstrating the algorithm’s stability and consistency across different tools. While each distance metric offers a unique perspective, their performance may also be influenced by the type of criteria involved. For example, in scenarios where quantitative measures dominate, Euclidean-based metrics might yield more interpretable rankings. This observation reinforces the need for future studies to investigate metric-criteria alignment more systematically.

### Realistic sensitivity analysis with data perturbation

In addition to the variance-based sensitivity analysis, we conducted a perturbation-based sensitivity test to evaluate the robustness of the final ranking against small fluctuations in the data—a realistic concern in decision-making problems based on subjective expert assessments. For this purpose, each of the mean distance values in Table 3 was perturbed by up to ±7%, simulating potential imprecision or noise in the input. By performing 100 Monte Carlo simulations, where the perturbed distances were used to re-rank the alternatives. The analysis revealed that the low ranking and top ranking alternatives remained unchanged in 85% and 93% of the simulations, respectively. This confirms that the decision-making model is resilient to small input disturbances and supports the reliability of the results.

**Table 6:** Stability of Alternative Rankings.

**Alternative**	**Mean Distance**	**Frequent Rank**	**Stability (%)**
$\varsigma _1$	1.12315	4	85%
$\varsigma _2$	1.23120	2	80%
$\varsigma _3$	1.15010	3	88%
$\varsigma _4$	1.25375	1	93%

## Comparative study

The site selection of warehouses or distribution centers has been widely explored in numerous research articles. Some recent studies^24–27^ are based on mathematical frameworks (see also^28,29^), while others^30–33^ rely on theoretical approaches. The proposed model, which leverages the CPFSS-a generalized structure combining fuzzy systems and soft set theory-offers distinct advantages due to its hybrid and flexible nature. In particular, the complex Pythagorean fuzzy soft set is well-suited for handling large datasets, as its structure accounts for the periodicity of each data point within the complex plane. This makes the proposed model more robust and effective compared to the methods introduced in^30–33^, which lack the ability to perform compact numerical calculations for dependent attribute data. By contrast, the proposed model provides a comprehensive mathematical framework for identifying the optimal site for warehouse locations. For a meaningful comparison, the proposed model can be evaluated alongside existing mathematical approaches discussed in the recently published works^24–27^. The Table 7 shows the results according to the existing models and proposed model.

**Table 7:** Comparison of proposed algorithm with the existing algorithms.

Models	Ranking
Saha et. al^24^	$R (\grave{\mathcal {H}}(\varsigma _{1}))\le R (\grave{\mathcal {H}}(\varsigma _{4}))\le R (\grave{\mathcal {H}}(\varsigma _{2}))\le R (\grave{\mathcal {H}}(\varsigma _{3}))$
Ayyildiz et. al^25^	$R (\grave{\mathcal {H}}(\varsigma _{1}))\le R (\grave{\mathcal {H}}(\varsigma _{4}))\le R (\grave{\mathcal {H}}(\varsigma _{3}))\le R (\grave{\mathcal {H}}(\varsigma _{2}))$
Zhang, H., & Wei, G.^26^	$R (\grave{\mathcal {H}}(\varsigma _{1}))\le R (\grave{\mathcal {H}}(\varsigma _{4}))\le R (\grave{\mathcal {H}}(\varsigma _{3}))\le R (\grave{\mathcal {H}}(\varsigma _{2}))$
Demir et. al^27^	$R (\grave{\mathcal {H}}(\varsigma _{1}))\le R (\grave{\mathcal {H}}(\varsigma _{3}))\le R (\grave{\mathcal {H}}(\varsigma _{2}))\le R (\grave{\mathcal {H}}(\varsigma _{4}))$
Proposed	$R (\grave{\mathcal {H}}(\varsigma _{1}))\le R (\grave{\mathcal {H}}(\varsigma _{3}))\le R (\grave{\mathcal {H}}(\varsigma _{2}))\le R (\grave{\mathcal {H}}(\varsigma _{4}))$

**Figure 5:**
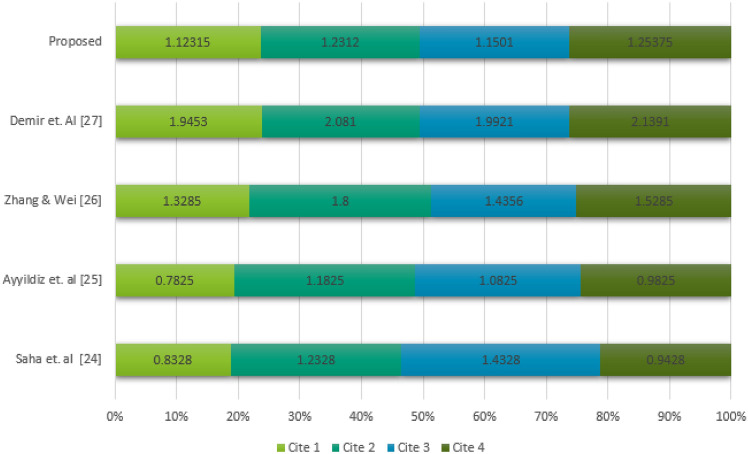
Comparative analysis.

From Table 7, it is evident that the site $\varsigma _{1}$ is the most suitable selection for the warehouse. The comparison presented in this table also demonstrates that the results of the proposed algorithm are both acceptable and reliable when evaluated against existing models. This reliability stems from the fact that the proposed algorithm is distance-dependent on the structure of the complex Pythagorean fuzzy soft set, which represents a more advanced and comprehensive framework compared to existing structures. Consequently, the proposed model is not only more sophisticated but also broader in scope than the current alternatives. The proposed model demonstrates greater accuracy and adaptability compared to existing models, as it utilizes the advanced structure of the complex Pythagorean fuzzy soft set. Unlike existing algorithms, which lack comprehensive distance-based evaluations, the proposed approach offers a more robust framework for optimal site selection. A graphical comparison of proposed structure with existing model with calculating values is shown in Fig. 5.

### Managerial implication

The proposed model equips supply chain and logistics managers with a robust and flexible decision-support tool for warehouse site selection under uncertainty. By incorporating the CPFSS framework, the model enables decision-makers to account for hesitation and imprecision in expert evaluations-common issues in real-world strategic planning. The integration of multiple distance measures provides a multi-faceted view of each alternative, promoting more reliable site selection. Additionally, the model’s ability to withstand minor fluctuations in input data, as demonstrated through perturbation-based sensitivity analysis, assures managers of its practical stability and consistency. These features make the model especially suitable for complex, high-stakes environments where decision accuracy and robustness are critical. Managers can also adapt this approach for broader applications such as facility expansion, vendor evaluation, and risk-sensitive investment decisions.

## Conclusion

This paper is the extension of the Pythagorean fuzzy soft set to CPFSS. In this article, the fundamentals of the CPFSS are introduced and also illustrated with the help of examples. Further the distance measures between the CPFSS and the distance measures between the complex Pythagorean fuzzy soft elements are proposed with the proof of the distance measure properties. On the basis of the proposed distances, an algorithmic approach is also established which is then applied to the warehouse site selection case study for the validity and usefulness of the this approach. After this, a comprehensive comparison of proposed algorithmic approach with existing models is presented. This comparison point-out the advancements of the presented model, specially in terms of its versatility, applicability and robustness in dealing with decision-making scenarios with complex data. The results demonstrate that the proposed algorithmic approach is capable of producing robust and interpretable outcomes across various distance metrics. While the top-ranked alternative remains consistent with existing methods, our model reveals nuanced distinctions—particularly in the evaluation of Location 4—which may offer improved decision support in complex environments. Its uses to various decision-making scenarios and the ability to tackle the uncertainty make this approach a valuable technique for practical applications in the medical field, as well as other fields which requires complex decision-making models. Moreover, the divergence analysis revealed that the Euclidean distance produced the most distinct rankings compared to the others, suggesting it introduced greater variability into the aggregated result. This highlights the importance of understanding metric behavior in multi-metric decision models. Future research may consider the selective use of distance metrics based on the nature of individual criteria, enabling more tailored and potentially more accurate decision outcomes. Although the proposed method demonstrates flexibility and robustness in the context of warehouse site selection, several limitations should be acknowledged. First, the study applies each distance metric uniformly across all criteria, regardless of the nature of the data—an approach that may overlook opportunities for metric-specific optimization. Second, the method depends on expert assessments, which, while practical, introduce a degree of subjectivity that may affect reproducibility. Future work will address these aspects to enhance both the reliability and applicability of the method. Additionally, the current study is confined to logistics-based site selection, the underlying framework has the potential to be adapted for other domains, such as financial decision-making or medical diagnostics. However, such extensions require domain-specific modeling and validation, which we identify as promising directions for future research.

## Data Availability

All data generated or analysed during this study are included in this published article [and its supplementary information files].
